# The Effect of Mycotoxins and Silymarin on Liver Lipidome of Mice with Non-Alcoholic Fatty Liver Disease

**DOI:** 10.3390/biom11111723

**Published:** 2021-11-19

**Authors:** Kamila Bechynska, Vit Kosek, Marie Fenclova, Lucie Muchova, Vaclav Smid, Jakub Suk, Karel Chalupsky, Eva Sticova, Kamila Hurkova, Jana Hajslova, Libor Vitek, Milena Stranska

**Affiliations:** 1 Department of Food Chemistry and Analysis, University of Chemistry and Technology, 166 28 Prague, Czech Republic; bechynsk@vscht.cz (K.B.); kosekv@vscht.cz (V.K.); fenclovm@vscht.cz (M.F.); hurkovak@vscht.cz (K.H.); hajslovj@vscht.cz (J.H.); 2 Institute of Medical Biochemistry and Laboratory Diagnostics, General University Hospital and 1st Faculty of Medicine, Charles University, 128 08 Prague, Czech Republic; lucie.muchova@centrum.cz (L.M.); j.suk@email.cz (J.S.); vitek@cesnet.cz (L.V.); 3 4th Department of Internal Medicine, General University Hospital and 1st Faculty of Medicine, Charles University, 128 00 Prague, Czech Republic; Vaclav.smid@lf1.cuni.cz; 4 Institute of Molecular Genetics of the Czech Academy of Sciences, 142 20 Prague, Czech Republic; krlcha@img.cas.cz; 5 Institute for Clinical and Experimental Medicine, 140 21 Prague, Czech Republic; evsc@ikem.cz

**Keywords:** mycotoxins, silymarin, lipidome, metabolome, mice liver, mass spectrometry

## Abstract

Milk thistle-based dietary supplements have become increasingly popular. The extract from milk thistle (*Silybum marianum*) is often used for the treatment of liver diseases because of the presence of its active component, silymarin. However, the co-occurrence of toxic mycotoxins in these preparations is quite frequent as well. The objective of this study was to investigate the changes in composition of liver lipidome and other clinical characteristics of experimental mice fed by a high-fat methionine-choline deficient diet inducing non-alcoholic fatty liver disease. The mice were exposed to (i) silymarin, (ii) mycotoxins (trichothecenes, enniatins, beauvericin, and altertoxins) and (iii) both silymarin and mycotoxins, and results were compared to the controls. The liver tissue extracts were analyzed by ultra-high performance liquid chromatography coupled with high-resolution tandem mass spectrometry. Using tools of univariate and multivariate statistical analysis, we were able to identify 48 lipid species from the classes of diacylglycerols, triacylglycerols, free fatty acids, fatty acid esters of hydroxy fatty acids and phospholipids clearly reflecting the dysregulation of lipid metabolism upon exposure to mycotoxin and/or silymarin.

## 1. Introduction

Mycotoxins are secondary metabolites produced by fungi that contaminate various foods consumed by humans. The mycotoxins frequently occurring in herbs and plant-based foods include mainly the group of trichothecenes, zearalenones, enniatins and beauvericin (produced by *Fusarium* sp.), as well as the groups of toxins produced by *Alternaria*, *Penicillium* or *Aspergillus* sp. [1]. These mycotoxins show different toxicological effects. For trichothecenes, the common manifestations are suppression of immune system, nausea and vomiting, associated with inhibition of DNA/RNA and protein synthesis [2], hepatotoxicity and carcinogenicity [3]. Zearalenone and its derivatives also negatively affect the DNA and protein synthesis, and have estrogenic activities in various animal species [2]. For enniatins and beauvericin, the cytotoxicity includes deterioration of ionic homeostasis and increase of the cell membrane permeability [4]. In regard to the Alternaria toxins, they cause mutagenicity, genotoxicity and carcinogenicity [5], as in the case of other mycotoxins produced by *Aspergillus* and *Penicillium* fungi.

Exposure to mycotoxins is a real global problem with substantial and widespread health threats [6]. In fact, several reports have described mycotoxicosis outbreaks as a result of mycotoxin contamination across the world [7,8,9]. Although direct toxic effects of individual mycotoxins are relatively well known [10,11], there is still a lack of knowledge on the effects of mixtures of variously co-occurring mycotoxins. From the numerous studies focused on characterization of mycotoxins’ toxicity in the in vivo animal studies, only several of them use the metabolomics-based approach. Liu et al. investigated the effect of zearalenone (ZEA) supplementation on metabolism of rats (3 mg/kg body weight (b.w.) per day for two weeks), and the urine and plasma metabolome was investigated by the NMR-based metabolomics. The results of the study demonstrate the changes in concentrations of some polar metabolites (such as lactate, glucose, amino acids, ketones and glycoproteins) suggesting that zearalenone exposure causes the oxidative stress and changes common systemic metabolic processes, including cell membrane metabolism, protein biosynthesis, and glycolysis [12]. The studies of Ji et al. were focused on assessment of toxic effects of deoxynivalenol (DON) and ZEA in mice liver, serum and urine after administration of 2 mg/kg b.w. DON and 20 mg/kg b.w. ZEA, both individually and in combination. The metabolomics profiles showed that DON and ZEA have antagonistic effect, and the metabolic pathway analysis demonstrated that the combined DON and ZEN treatment could downregulate the valine, leucine and isoleucine biosynthesis, glycine, serine and threonine metabolism, and changes in representation of O-glycosyl compounds demonstrated the changes in glucose metabolism [13,14]. Detailed metabolomic studies become even more important considering the fact that, based on the reported studies, not only the absolute concentration of any single mycotoxin but also their co-occurrence on lower concentration levels could result in substantial health threats [15,16,17,18].

The co-occurrence of mycotoxins is frequent especially in herbal-based dietary supplements [19], with *Silybum marianum* sp. plant (milk thistle) as one of the main contributors [1]. The major health-beneficial component represented in milk thistle dietary supplements is silymarin, a complex of flavonolignans and flavonoids represented by silybin A/B, isosilybin A/B, silydianin, silychristin, isosilychristin, taxifolin, and approx. 30% of undefined yet polymeric (polyphenolic) fraction [20]. Despite the fact that the effects of silymarin against mycotoxins have been examined in several in vivo animal studies (ochratoxin A [21], fumonisin B1 [22], aflatoxins [23,24,25] and ZEA with DON or T-2 toxin [26,27]), any unambiguous statements about its antagonistic effect have not been concluded so far. It is worth noticing that among the used methodologies, i.e., assessment of the growth performance, weight gain and feed intake, histological and serum biochemical analysis, the metabolomics approach has not been used by any group.

Since silymarin is worldwide used for its putative hepatoprotective effects, the aim of this study was to investigate the effect of silymarin and/or mycotoxins on changes in composition of liver lipidome of mice fed by high-fat methionine-choline deficient (MCD) diet to mimic the most frequently occurring noxious environment in a human food chain. Hence, non-alcoholic fatty liver disease (NAFLD) was induced in mice, as they should serve as a model simulating the patients with the liver disease, who use silymarin-based dietary supplements as a part of their medical treatment. A mixture of 13 mycotoxins was designed based on results of the previous authors’ study [1] revealing trichothecenes (T-2 toxin, HT-2 toxin, deoxynivalenol, diacetoxyscirpenol), enniatins (enniatin A, enniatin A1, enniatin B, enniatin B1), beauvericin, zearalenone, and altertoxins (alternariol, alternariol monomethyl ether, tentoxin) to be frequently present in silymarin-containing dietary supplements. To our knowledge, this is the first study focusing on the non-polar metabolome of liver with respect to hepatoprotectivity and hepatotoxicity of silymarin and mycotoxins, respectively.

## 2. Materials and Methods

### 2.1. Standards and Chemicals

The standards of mycotoxins were purchased from Sigma-Aldrich (Darmstadt, Germany). Stock solutions of T-2 toxin (T-2), HT-2 toxin (HT-2), diacetoxyscirpenol (DAS), deoxynivalenol, beauvericin (BEA), zearalenone, enniatin A (ENN-A), enniatin A1 (ENN-A1), enniatin B (ENN-B), enniatin B1 (ENN-B1), alternariol (AOH), alternariol monomethyl ether (AME), and tentoxin (TEN) were prepared in methanol and maintained at −20 °C in dark. Silymarin (defined as ’flavonolignan mixture extracted from the seeds of *Silybum marianum*’, product number S0292, Lot BCBM3466V, declared content of silybin A/B 42.6%), was purchased from Sigma-Aldrich (Darmstadt, Germany). The concentrations of individual silymarin components as quantified in our previous study [28] were as follows: 25.3 ± 0.8 mg/g of taxifolin, 110 ± 3.4 mg/g of silychristin, 32 ± 0.9 mg/g of silydianin, 113 ± 3.1 mg/g of silybin A, 133 ± 3.9 mg/g of silybin B, 67.4 ± 2.4 mg/g of isosilybin A, 26.1 ± 1.1 mg/g of isosilybin B, 5.0 ± 0.3 of 2,3-dehydrosilybin. The stock solution of silymarin was prepared in 40% polyethylene glycol (PEG 400, Sigma Aldrich, Darmstadt, Germany) solution. All of the HPLC-grade solutions (methanol, ethanol, methyl tert-butyl ether, 2-propanol, acetonitrile) and mobile phase modifiers (ammonium acetate, ammonium formate, acetic acid, formic acid) were purchased from Sigma-Aldrich (Darmstadt, Germany). Deionized water (dH2O) was obtained from a Milli-Q^®^ Integral system (Millipore supplied by Merck, Darmstadt, Germany).

### 2.2. Animals and In Vivo Study Design

For the in vivo testing, inbred male mice C57B1/6J, 10–12 weeks old were used (*n* = 8 per experimental group). The animals were housed under controlled temperature and a natural 12: 12 light–dark cycle. In the first six weeks, mice were exposed to high-fat MCD diet (ssniff-Spezialdiaten, Soest, Germany) to induce NAFLD. In the next three weeks, mixtures of silymarin and mycotoxins were administered to mice by intragastric tube on daily basis.

The administered doses of mycotoxins and silymarin are summarized in Table 1. The spectrum of mycotoxins in the experiment was settled to simulate the exposure after consuming of milk thistle-based dietary supplements (as we determined in our previous study, [1], and as part of the “proof of principle” study design, we used 1000× higher concentration levels. The administered dose for silymarin was 30 mg/kg b.w. per day, previously proved as an effective dose in similar study design [29]. More details to the experimental mycotoxins mixture and re-calculations are presented in Table S1.

The experimental group ‘B2′ was treated with mixture of mycotoxins only, the group ‘C3’ was treated with silymarin only, the group ‘D4’ both mycotoxins and silymarin and ‘A1’ was the control group. The basic mixture of mycotoxins was prepared in ethanol, and before administration, it was diluted into 40% polyethylene glycol (PEG) (solvent used for dissolving of silymarin) to final concentration of 1% of ethanol. The ratio of solvents administered to animals (i.e., water: PEG: ethanol in 59.4: 39.6: 1, *v/v/v*) was the same in all of the experimental variants, including the control group.

At the end of the experiment, mice were anesthetized with 4% isoflurane (0.3 L/min flow), blood was sampled from the ocular vein and the animals were sacrificed by cervical dislocation. Liver was removed, weighed and sampled for histological and lipidomic analyses. After blood collection, each sample was kept for 10 min at room temperature and then on ice until centrifugation. Samples were centrifuged within 1 h of collection at 5000× *g* for 10 min at 4 °C. Separated sera were frozen at −80 °C.

All aspects of the study met the accepted criteria of experimental use of laboratory animals and all protocols were approved by the Animal Research Committee of the 1st Faculty of Medicine, Charles University, Prague, Czech Republic (MSMT 12181/2016-5 from 5.4.2016).

### 2.3. Laboratory Analyses

Serum biochemical markers (glucose, alanine aminotransferase (ALT), aspartate aminotransferase (AST), alkaline phosphatase (ALP), lactate dehydrogenase (LD), total and HDL cholesterol, and triacylglycerols (TGs)) were determined by standard assays using an automatic analyzer (Modular analyzer, Roche Diagnostics GmbH, Mannheim, Germany). Bilirubin concentrations in the serum and liver tissue were determined as described previously [29]. The results of laboratory analyses are presented in Table 2.

### 2.4. Liver Histology

Two small liver tissue blocks (about 1 cm^3^) were fixed in 4% paraformaldehyde followed by a standard procedure for paraffin embedding. Serial sections 5–7 µm thick were cut a stained with haematoxylin and eosin, and Verhoeff’s van Gieson stain. Each slide was viewed using standard light microscopy. Histological assessment was performed according to Kleiner et al. [30]. In brief, each representative section from the tissue blocks was blindly assessed for NAS score (steatosis, ballooning, intralobular inflammation), the ratio of micro/macrovesicular steatosis, portal and periportal inflammatory infiltrates, Mallory-Denk bodies, and signs of fibrosis.

### 2.5. Sample Preparation for Lipidomic Analyses

Liver tissue samples were first frozen at −80 °C and then lyophilized. Then, 25 mg of the powder was weighed into FastPrep tubes with lysing matrix D, 1.4 mm ceramic spheres (MP Biomedicals, Santa Ana, CA, USA) and 1 mL of methyl tert-butyl ether: methanol (MTBE: MeOH, 3: 1, *v/v*) was added. These were extracted 4 × 15 s, the tubes were shaken vigorously between each of the homogenization runs to ensure proper distribution of the sample. Then, 450 µL of dH_2_O per 25 mg of powder was added to induce phase separation. After vortexing for 10 s, the tubes were centrifuged at 14,000 rpm (21,255× *g*). Five hundred µL of the upper organic phase was transferred to an Eppendorf microtube Fisherbrand^™^ (Thermo Fisher Scientific, Waltham, MA, USA), evaporated to dryness and stored at −80 °C until analysis. Prior to analysis, the stored samples were resuspended in 500 µL iPrOH: MeOH: H_2_O (65: 30: 5, *v/v/v*). After centrifugation, 450 µL of the solution was transferred to a vial for ultra-high performance liquid chromatography coupled with high-resolution tandem mass spectrometric (U-HPLC-HRMS/MS) analysis. All samples were measured in duplicates (in randomized order). To assure the quality of U-HPLC-HRMS/MS measurements, the quality control (QC) samples were prepared (by transferring of 25 µL of each sample into the 4 mL vial), and run together with all of the samples.

### 2.6. Lipidomic U-HPLC-HRMS/MS Analysis

For the lipidomic analysis, U-HPLC (Infinity 1290, Agilent, Santa Clara, CA, USA) coupled to a high-resolution mass spectrometer with a hyphenated quadrupole time-of-flight mass analyzer (6560 Ion Mobility Q-TOF LC/MS; Agilent, Santa Clara, CA, USA) with the Agilent Jet Stream electrospray (ESI) source were employed.

For chromatographic separation of sample components, Acquity BEH C18 (1.7 μm, 2.1 mm × 150 mm (Waters, Milford, MA, USA) was used. The chromatographic system used with ESI+ detection was: A-10 mM ammonium formate and 0.1 % formic acid in acetonitrile: water (60: 40, *v/v*); B-10 mM ammonium formate and 0.1 % formic acid in 2-propanol: acetonitrile (90: 10, *v*/*v*). For chromatographic separation of liver detected in ESI-mode, following mobile phases were used: A-10 mM ammonium acetate and 0.1% acetic acid in acetonitrile: water (60: 40, *v/v*); B-10 mM ammonium acetate and 0.1% acetic acid in 2-propanol: acetonitrile (90: 10, *v/v*). The flow rate was constant at 0.3 mL min^−1^. The column temperature was maintained at 60 °C, the injection volume was increased to 1 μL in ESI+ mode and 5 μL in ESI- mode. The autosampler was kept at 10 °C.

The mass analyzer was operated at the following conditions at both ionization modes: Gas temperature 350 °C, Drying Gas 12 L/min, Nebulizer pressure 40 psi, sheath gas temperature 350 °C, sheath gas flow 11 L/min, nozzle voltage 250 V, fragmentor voltage 380 V, octopole radiofrequency voltage 750 V. The capillary voltage was 3000 V in ESI+, 4000 V in ESI-. For all samples, the data were acquired over the *m/z* range of 100–1700 at the rate of 1 spectrum/s. The *m/z* range was autocorrected on reference masses 121.0509 and 922.0098 for positive mode and 119.0363 and 980.0164 in negative mode. To obtain the fragmentation spectra of lipids, the QC sample was run several times in auto MS/MS mode with collision energies 10, 20 and 40 eV, with acquisition rate of 5 spectra/s for full spectra and 7 spectra/sec for fragmentation spectra. Top 5 ions were chosen for fragmentation with the dynamic exclusion for 0.1 min after fragmentation event.

### 2.7. Lipid Identification and Statistical Analyses

The mass spectrometry data were processed in LipidMatch suite [31] which uses MZmine 2 for feature extraction and an R script for lipid identification based on in silico fragmentation databases. At least a class-specific fragment was required for lipid identification. When the other specific fragments were available, lipid species could be characterized by the fatty acids present (i.e., by number of carbons and double bonds in the non-polar chain). All lipids were identified based on MS/MS spectra obtained by repeated fragmentation of QC sample with different collision energies.

Lipidomic data processing was performed in both web-based and R based MetaboAnalyst (metaboanalyst.ca) packages followed by SIMCA (Sartorius, Göttingen, Germany). The data were firstly overviewed with principal component analysis (PCA) in MetaboAnalyst, where sum normalization, log transformation and Pareto scaling were employed for MS signals processing in order to ensure normal distribution and higher significance of low abundant compounds. Statistically insignificant compounds not complying the criteria of Analysis of Variance (ANOVA) *p*-value < 0.01 (False Discovery Rate (FDR) adjusted) were filtered out, and the filtered data matrix was normalized by sum in MS Excel (where also the fold change values were calculated), and then loaded to SIMCA. When binary orthogonal partial least squares discriminant analysis (OPLS-DA) models were built, logarithmic transformation and Pareto scaling were further applied. The significant lipid species were selected based on their Variable Importance in Projection (VIP) score in binary OPLS-DA models and their significance between the groups was evaluated according to the fold change and ANOVA Fisher post hoc test. Additionally, Pattern Hunter in MetaboAnalyst using Spearman’s rank correlation coefficient was applied to confirm the trends of increasing intensities of lipids between treated groups.

## 3. Results

### 3.1. The Effect of Mycotoxin Exposure and Silymarin Treatment on Clinical and Laboratory Characteristics of Mice Fed High-Fat MCD Diet

Basic anthropometric parameters (body and liver weight) did not differ among the experimental groups and no difference was observed also for main metabolic parameters including serum and liver bilirubin concentrations, liver enzyme activities, blood lipids and glycemia (Table 2). High-fat MCD diet led to development of liver steatosis in all experimental groups in the extent of 5–50% of hepatocytes with no significant differences among the groups. Only focal mild portal-based inflammation and low grade lobular necro-inflammatory activity was detected in histological samples with no evidence of liver cell ballooning degeneration and fibrosis. Detailed results on liver histology can be seen in Figure S1 of the Supplementary Materials.

### 3.2. Liver Lipidome Analysis

**Figure 1:**
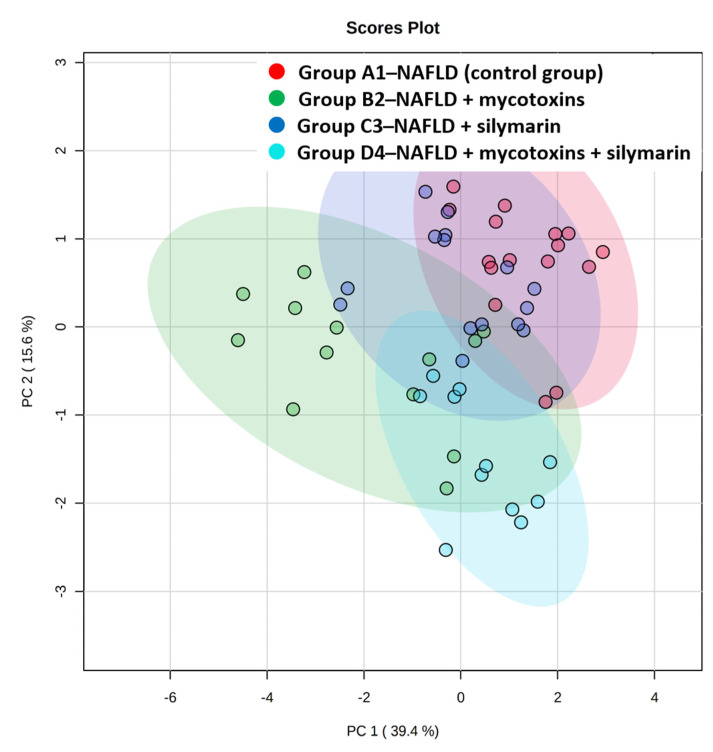
PCA score plot of 92 MS/MS confirmed lipids filtered out by ANOVA FDR *p*-value < 0.01.

Despite the fact that no significant changes in anthropometric, laboratory and histological parameters were detected among experimental groups, there were substantial changes in the liver lipidome. Out of 4235 mass spectrometric features detected in the samples, 344 were MS/MS confirmed lipid signals (after removing duplicates of the same lipids but differently ionizing adducts), and these were used for statistical evaluation. Before multivariate analysis, ANOVA was performed in order to reduce the number of features in samples and to select significant lipids which contributed to discrimination between groups. Based on FDR *p*-values < 0.01, 92 lipids significantly differing between at least two groups were filtered out for further analysis. The detailed characterization of these lipid species, i.e., the retention time of chromatographic separation and the *m/z* measured, is given in Table S2. The most frequent fatty acids in these 92 lipids were C16 (in particular, C16: 0 and C16: 1 represented in 15 and 2.5% lipid species, respectively), C18 (C18: 0, C18: 1, C18: 2 and C18: 3 in 10, 19, 16 and 6% lipid species, respectively), C20 (C20: 1, 20: 3 and 20: 4 represented in 4, 4, and 7% lipid species, respectively), and C22 (C22: 1, C22: 3, C22: 4, C22: 5 and C22: 6 in 1.2, 1.2, 1.2, 1.9 and 7.5% lipid species, respectively). It should be noticed that this calculation could be realized only from lipid species providing MS/MS fragments referring to particular fatty acids, i.e., for 76 out of 92 lipid species, so the presented values may not be entirely accurate, but we do not assume a significant bias. The PCA score plot is depicted in Figure 1 and illustrates evident changes in lipidome between the mycotoxin- and silymarin-exposed mice groups. The largest difference seems to be observable between group B2 (administered by mycotoxins only) and control group (group A1). The fold changes expressing the differences between intensities of signals of particular lipid features between the groups are illustrated in Table S2.

**Figure 2:**
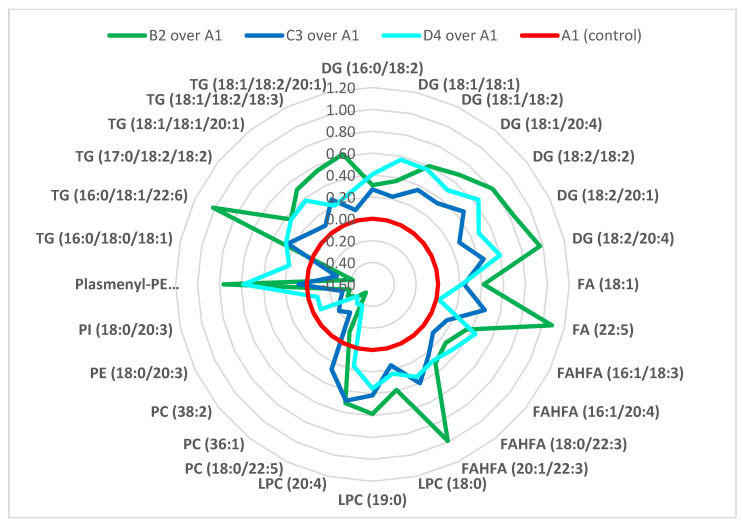
The radar chart of 28 significantly elevated lipids upon exposure to mycotoxins and/or silymarin. Data are expressed as log2(FC) over control. (Lipids with OPLS-DA VIP score > 1 significantly contributing to the group separation in at least two binary comparisons are stated).

As the next step, three binary OPLS-DA models were built to identify the sets of the most significant lipids having the greatest influence on separation of the experimental groups. Groups B2, C3 and D4 were consecutively compared with the control group (B2 vs. A1, C3 vs. A1 and D4 vs. A1), and all lipids with OPLS-DA VIP score > 1 were selected. The three partially overlapping subsets of 38, 29 and 33 lipids for ‘B2 vs. A1′, ‘C3 vs. A1′ and ‘D4 vs. A1′, respectively, are summarized in Tables S3–S5. From these sets, 28 lipids that significantly contributed to groups separation in at least two binary comparisons were selected. The fold changes of these lipids between the particular sample groups are summarized in Table 3, and depicted in the Figure 2 radar chart (in the Figure S2, boxplots for all of these lipids are illustrated). As we can observe from the Table 3 and Figure 2, the majority of fatty acids present in filtered statistically significant lipids were of 18 carbons and more, in particular C18: 1, C18: 2, C18: 3, C20: 4, C22: 3, C22: 5 and C22: 6. 

The intervention of silymarin/mycotoxins administration caused upregulation of triacylglycerols (TGs), diacylglycerols (DGs), free fatty acids (FAs), fatty acid esters of hydroxyl fatty acids (FAHFAs), lysophosphatidylcholines (LPCs) and one plasmenyl-phosphatidylethanolamine. The opposite trend was visible only for phospholipids from the group of phosphatidylcholines (PCs), phosphatidylethanolamines (PEs) and phosphatidylinositols (PIs).

The most pronounced effect was obvious for a group of seven DGs. All of them were significantly increased in both groups B2 and D4 (where mycotoxins were administered) compared to the control group. The intensities were 1.3–1.55× higher in group D4 (mycotoxins + silymarin) and 1.5–2× higher in mycotoxins B2 group, where no protective effect of silymarin can be expected. The situation was very similar for TGs with significantly upregulated levels (1.53–2×) after administration of mycotoxins only (group B2), and only slight upregulation after mycotoxins and silymarin combined administration (group D4). The levels of both TGs and DGs stayed unchanged after silymarin administration when compared to the control group.

In case of FAHFAs, there was a significant upregulation in mycotoxins administered group B2, and less significant upregulation for group D4 (mycotoxins + silymarin), whereas for group C3 (silymarin), the FAHFA levels stayed almost the same when compared to control. Both FAs, oleic acid (FA (18: 1)) and docosapentaenoic acid (FA (22: 5), were significantly increased in mycotoxins administered group only. A similar trend was observed for LPCs, where all three LPC species significantly contributing to separation of experimental groups showed the highest intensity in the group B2 (mycotoxins administered mice).

As concerns the downregulated phospholipids (PLs), their intensity (except for PC (18: 0/22: 5)) was the lowest in mycotoxins administered B2 group, with a significant intensity drop (about 35%) when compared to the control. Less pronounced downregulation was observed in silymarin administered group (group C3) and group administered by both silymarin and mycotoxins (D4).

To summarize the results described above, all upregulated lipids were the most intensive in group B2 and most of them (TGs, DGs, FAHFAs) showed a repeating trend of increasing intensity in A1→C3→D4→B2 direction (i.e., from the control group, over the silymarin treated group, silymarin/mycotoxins treated group, to the group administered by mycotoxins). To confirm this observed trend between the experimental groups (Figure 2 and Table 3), a complementary statistical tool “Pattern hunter” looking for lipids correlating with specific intensity trends between the groups was applied. The list of the lipid species correlating with A1→C3→D4→B2 pattern is presented in Table 4. It is worth noticing that in addition to this trend, also another logical trend reflecting the positive effect of silymarin and negative effect of mycotoxins (i.e., C3→A1→D4→B2) was examined, and the corresponding lipid species are listed in Table 5.

As can be seen from Table 4, lipids reflecting a changed lipidome after exposure to silymarin, mycotoxins, and both silymarin and mycotoxins (A1→C3→D4→B2 pattern) were (plasmenyl)-phospholipids, TGs and FAHFA, which confirmed the results presented in Table 3 and Figure 2.

The new interesting data were obtained by hunting the pattern C3→A1→D4→B2, reflecting the contradictory effect of silymarin (on one side) and mycotoxins (on the other side, when compared to control). In addition to plasmenyl-phospholipids, phospholipids and TG, the new lipid group, ceramides (Cer), were identified to be downregulated in the silymarin administered group and upregulated in both groups with mycotoxin exposure (see Table 5).

## 4. Discussion

NAFLD and subsequent development of non-alcoholic steatohepatitis (NASH) belongs to very common hepatic manifestation of the metabolic syndrome, characterized by steatosis, hepatocytes damage, inflammation and fibrosis, which increase the risk of development of liver cirrhosis and even hepatocellular carcinoma [32]. The popularity of silymarin for treatment of liver diseases has been increasing, and extract of milk thistle, *Silybum marianum*, is among the most common herbal-based dietary supplements in the US [33]. Although most experimental reports as well as some clinical data suggest it does play a beneficial role [34,35,36], silymarin is generally considered to have negligible clinical importance [37,38,39,40]. In addition to the lack of properly controlled clinical trials, and poor definition of silymarin preparations used in such clinical studies [20], the other limitations include possible presence of biological contaminants of these preparations [41,42,43], such as mycotoxins [1,19,44,45].

Data of our current study assessing the non-polar liver extracts of silymarin/mycotoxin-exposed mice, demonstrated dynamical changes of the liver lipidome, in response to dietary intake of these groups of compounds. Quite surprisingly, the changes in hepatic lipidome of the mycotoxin/silymarin exposed groups were observed despite no apparent changes in basic anthropometric, laboratory and histological parameters occurred, suggesting that modulation of hepatic lipidome may precede changes in clinical parameters. This conclusion is supported by relatively short duration of dietary intervention which was not sufficient to substantially change clinical findings. On the other hand, lipidomic fingerprints have been proven to have enormous diagnostic potential in differentiating various stages of non-alcoholic fatty liver disease (NAFLD) including NASH [46,47] and it is likely that lipidomic analyses will have the role in diagnosing of other liver diseases as well.

It seems clear now that NAFLD is a complex disease with several subtypes differing in derangement of individual lipid metabolism pathways [48]. In fact, deterioration of metabolism of TG and DG [49], phospholipids [50], ceramides and sphingomyelines [51,52,53,54] was reported in NASH experimental animal models as well as human patients. Our observations fit into these previously reported results, as exemplified for instance by changes in metabolism of ceramides. Ceramides are members of the sphingolipid family taking part in the formation of the lipid bilayer of the cell membranes. Their formation has been associated with increased oxidative stress within the organism [32]. As toxicity of some of the mycotoxins used in our study, namely T-2, DON, ZEA and BEA, have been related to the increased production of free radicals and reactive oxygen species [55,56,57,58], our results demonstrating upregulation of ceramides in both mycotoxin-exposed groups, and their downregulation in silymarin treated group, are consistent with these previously reported data.

## 5. Conclusions

To conclude, this in vivo animal study confirms the effects of silymarin and mycotoxins exposure on complex hepatic lipidome of mice fed by high-fat MCD diet. The most important findings being revealed in this study are summarized below:

▪ Exposure to mycotoxins and silymarin, frequently occurring together in the dietary supplements intended for liver disease treatment, plays a significant role in the lipid metabolic response, which occurs earlier than the clinical manifestation.
▪ The majority of fatty acids present in lipid classes differentiating the experimental groups, i.e., groups of mice exposed to mycotoxins, mycotoxins + silymarin, and silymarin, were (poly)unsaturated fatty acids of 18 carbons and more, in particular C18: 1, C18: 2, C18: 3, C20: 4, C22: 3, C22: 5 and C22: 6.
▪ The administration of silymarin and/or mycotoxins led to changed metabolic regulation of TG, DGs, FAs, FAHFAs, LPCs, PCs, PIs, PEs, plasmenyl-PEs and ceramides, where ceramides, formation of which is closely associated with the oxidative stress, was the only lipid group unequivocally downregulated in silymarin administered groups, and upregulated in the groups where mycotoxins were fed.

Despite several limitations of the present study hindering more accurate interpretation of our observations (firstly, dietary intervention used in our study did not result in development of significant inflammatory changes in the liver tissue, most likely due to a relatively short period of intervention, and secondly, the control group with no dietary intervention was not used, as the study was designed to mimic the situation of the patients already suffering from a NAFLD/NASH and using the dietary supplements to treat or protect from these pathologies), the undertaken research brought interesting pilot results. To confirm our data, and improve the metabolomical–clinical understanding, further studies comprising, e.g., prolonged duration of the experiment, different dosages and methods of administration, are planned.

## Figures and Tables

**Table 1:** The administered doses of mycotoxins and silymarin in mg/kg b.w. and day.

Administered Compound		mg/kg b.w. and Day
Mycotoxins	AOH	0.16
	AME	0.06
	DON	0.15
	ZEA	0.01
	T-2	0.14
	HT-2	0.07
	DAS	0.001
	TEN	0.05
	BEA	0.09
	ENN-A	0.02
	ENN-A1	0.03
	ENN-B	0.07
	ENN-B1	0.04
silymarin		30

**Table 2:** The effect of mycotoxin exposure and silymarin treatment on clinical characteristics of mice fed high-fat MCD diet. Data expressed as mean ± SD or median and IQ range depending on their normality.

	Group A1 (Control)	Group B2 (Mycotoxins)	Group C3 (Silymarin)	Group D4 (Mycotoxins + Silymarin)	*p*-Value *
Body weight (g)	31.5 ± 3.2	29.7 ± 2.4	30 ± 4	31.9 ± 2.5	NS
Liver weight (g)	1.58 ± 0.19	1.3 ± 0.5	1.33 ± 0.2	1.48 ± 0.1	NS
ALT (U/L)	28 (23–41)	22 (15–35)	36 (17–46)	34 (32–43)	NS
AST (U/L)	90 (64–127)	58 (48–83)	66 (56–73)	71 (67–76)	NS
ALP (U/L)	85 ± 7.9	76 ± 9.6	83 ± 7	80 ± 12	NS
LD (U/L)	249 ± 76	220 ± 29	282 ± 114	251 ± 45	NS
Total cholesterol (mmol/L)	3 ± 0.5	2.5 ± 0.6	2.8 ± 0.4	3 ± 0.4	NS
LDL cholesterol (mmol/L)	0.64 ± 0.2	0.49 ± 0.2	0.56 ± 0.1	0.7 ± 0.1	NS
HDL cholesterol (mmol/L)	2.15 ± 0.3	1.89 ± 0.3	2.06 ± 0.3	2.3 ± 0.1	NS
Triacylglycerols (mmol/L)	0.62 ± 0.1	0.53 ± 0.1	0.59 ± 0.1	0.57 ± 0.1	NS
Glucose (mmol/L)	11.3 ± 1.4	12.2 ± 1	11.5 ± 1.9	12.3 ± 0.9	NS
Bilirubin in serum (μmol/L)	2.3 ± 0.8	2.3 ± 0.7	2.3 ± 0.1	2.2 ± 0.6	NS
Bilirubin in liver (μmol/g)	1.01 (0.8–1.1)	0.83 (0.7–2.2)	0.63 (0.6–0.9)	1.08 (0.7–1.2)	NS

* NS, non-significant when compared against control NAFLD group (A1).

**Table 3:** The effect of mycotoxin and silymarin exposure on liver lipid content. Lipid species with OPLS-DA VIP score > 1 significantly contributing to the group separation in at least two binary comparisons (B2, C3 and D4 vs. control) are stated. The fold changes are expressed as log2(FC), so the breakpoint between downregulation and upregulation is equal to 0.

	Fold Change (FC) Expressed as log_2_(FC)			
Lipid Name	B2 over A1	C3 over A1	D4 over A1	A1 (Control)
DG (16: 0/18: 2)	0.31 *	0.27 *	0.41 *	0
DG (18: 1/18: 1)	0.37 *	0.22	0.57 *	0
DG (18: 1/18: 2)	0.60 *	0.36 *	0.56 *	0
DG (18: 1/20: 4)	0.68 *	0.35	0.50 *	0
DG (18: 2/18: 2)	0.80 *	0.47 *	0.64 *	0
DG (18: 2/20: 1)	0.84 *	0.28	0.48 *	0
DG (18: 2/20: 4)	0.97 *	0.44 *	0.60 *	0
FA (18: 1)	0.42 *	0.24	0.22	0
FA (22: 5)	1.08 *	0.45	0.02	0
FAHFA (16: 1/18: 3)	0.35 *	0.16	0.44 *	0
FAHFA (16: 1/20: 4)	0.26 *	0.11	0.35 *	0
FAHFA (18: 0/22: 3)	0.32 *	0.22 *	0.29 *	0
FAHFA (20: 1/22: 3)	0.99 *	0.40	0.34	0
LPC (18: 0)	0.39 *	0.16	0.24	0
LPC (19: 0)	0.58 *	0.41 *	0.35 *	0
LPC (20: 4)	0.51 *	0.49 *	0.17	0
PC (18: 0/22: 5)	−0.12	0.26	−0.39 *	0
PC (36: 1)	−0.50 *	−0.27 *	−0.37 *	0
PC (38: 2)	−0.42 *	−0.21 *	−0.43 *	0
PE (18: 0/20: 3)	−0.35 *	−0.27 *	−0.08	0
PI (18: 0/20: 3)	−0.39 *	−0.32 *	−0.08	0
plasmenyl-PE (18: 1/18: 1)	0.76 *	0.08	0.58 *	0
TG (16: 0/18: 0/18: 1)	−0.42 *	−0.27 *	0.18	0
TG (16: 0/18: 1/22: 6)	1.02 *	0.26	0.28	0
TG (17: 0/18: 2/18: 2)	0.35 *	0.14	0.35 *	0
TG (18: 1/18: 1/20: 1)	0.51 *	0.09	0.38 *	0
TG (18: 1/18: 2/18: 3)	0.56 *	0.26	0.20	0
TG (18: 1/18: 2/20: 1)	0.62 *	0.10	0.27	0

* Difference between groups is statistically significant (*p* < 0.01) according to the Fisher post hoc test.

**Table 4:** The significantly changed lipid species correlating with A1→C3→D4→B2 pattern, based on Spearman correlation coefficients >0.5; B2, mycotoxin-exposed NAFLD group; C3, silymarin-treated NAFLD group; D4, mycotoxin plus silymarin-exposed NAFLD group; A1, control NAFLD group.

Lipid Name	Correlation
TG (16: 0/18: 2/18: 3)	positive
TG (16: 0/18: 3/18: 3)	positive
TG (16: 0/18: 3/18: 3) ^a^	positive
FAHFA (16: 1/20: 4 ) ^b^	positive
plasmenyl-PE (18: 1/18: 1) ^a,b^	positive
PC (34: 3) ^a^	positive
PE (34: 3)	positive
PE (36: 3)	positive
PE (38: 5)	positive
PC (16: 0/22: 6)	negative
PC (18: 2/22: 6)	negative
PC (18: 0/22: 5) ^b^	negative
PC (40: 8)	negative
PE (16: 0/22: 6)	negative
PE (18: 1/20: 4)	negative

^a^ correlating with both patterns A1→C3→D4→B2 and C3→A1→D4→B2. ^b^ having the OPLS-DA VIP score > 1 and significantly contributing to the group separation in binary comparisons (see Table 3).

**Table 5:** The significantly changed correlating with C3→A1→D4→B2 pattern, based on Spearman correlation coefficients >0.5; B2, mycotoxin-exposed NAFLD group; C3, silymarin-treated NAFLD group; D4, mycotoxin plus silymarin-exposed NAFLD group; A1, control NAFLD group.

Lipid Name	Correlation
Cer (18: 1/16: 0)	positive
Cer (18: 1/23: 0)	positive
Cer (18: 2/23: 0)	positive
Cer (25: 2/15: 0)	positive
TG (16: 0/18: 2/22: 6)	positive
TG (16: 0/18: 3/18: 3) ^a^	positive
TG (18: 1/18: 1/20: 1) ^b^	positive
TG (18: 1/18: 2/20: 1) ^b^	positive
TG (18: 2/18: 2/18: 3)	positive
DG (18: 2/20: 1) ^b^	positive
plasmenyl-PE (18: 1/18: 1) ^a,b^	positive
plasmenyl-PE (18: 1/20: 1)	positive
PC (34: 3) ^a^	positive
plasmenyl-PE (16: 0/22: 4)	negative

^a^ correlating with both patterns A1→C3→D4→B2 and C3→A1→D4→B2. ^b^ having the OPLS-DA VIP score > 1 and significantly contributing to the group separation in binary comparisons (see Table 3).

## Data Availability

Data are available upon request from the corresponding author.

## Supplementary Materials

The following are available online at https://www.mdpi.com/article/10.3390/biom11111723/s1, Table S1: Calculation of doses of particular mycotoxins in the study. Table S2: The fold changes for 92 lipid features by ANOVA. Table S3: The effect of mycotoxin exposure on hepatic lipid profile—features from binary OPLS-DA model (B2 vs. A1) with VIP score > 1. Table S4: The effect of mycotoxin exposure on hepatic lipid profile—features from binary OPLS-DA model (C3 vs. A1) with VIP score > 1. Table S5: The effect of mycotoxin exposure on hepatic lipid profile—features from binary OPLS-DA model (D4 vs. A1) with VIP score > 1. Figure S1: Liver steatosis staging: A-score 0 (steatosis of less than 5 % of liver cells), B-score 1 (steatosis of up to 33 % of liver cells), C-score 2 (steatosis of up to 66 % of liver cells), D-score 3 (steatosis of more than 66 % of liver cells). Figure S2: Boxplots for all 28 significant lipids with OPLS-DA VIP score > 1, significantly contributing to group separation in at least two binary comparisons.
