# Supplementary material for: The Effect of Mycotoxins and Silymarin on Liver Lipidome of Mice with Non-Alcoholic Fatty Liver Disease
Source: Biomolecules. 2021 Nov 19;11(11):1723. doi: 10.3390/biom11111723 (PMC8615755; doi:10.3390/biom11111723)
Supplement: Supplementary file 1 [file biomolecules-11-01723-s001.zip › biomolecules-1417725-supplementary.pdf]

Table S1. Calculation of doses of particular mycotoxins in the study.

Mycotoxin	Concentration of mycotoxins in milk thistle-based dietary supplement (ng/g)*	Concentration of mycotoxins considered for administration of mice (multiplied by 1000), ng/g	Producer's recommended daily dose (DD) of preparation		ng of mycotoxins in daily dose	ng of mycotoxins per kg of b. w. and day	ng of mycotoxins per mouse (0.03 kg) and day
			No of tablets*	Weight of the tablet (g)*			
**AOH**	6 834	6 834 000	**2**	**0.83**	11 344 440	162 063	**4 862**
**AME**	2 441	2 441 000			4 052 060	57 887	**1 737**
**DON**	6 477	6 477 000			10 751 820	153 597	**4 608**
**ZEA**	282	282 000			468 120	6 687	**201**
**T-2**	5 958	5 958 000			9 890 280	141 290	**4 239**
**HT-2**	2 985	2 985 000			4 955 100	70 787	**2 124**
**DAS**	36	35 973			59 716	853	**26**
**TEN**	2 127	2 127 000			3 530 820	50 440	**1 513**
**BEA**	3 891	3 891 000			6 459 060	92 272	**2 768**
**ENN-A**	722	722 000			1 198 520	17 122	**514**
**ENN-A1**	1 142	1 142 000			1 895 720	27 082	**812**
**ENN-B**	2 918	2 918 000			4 843 880	69 198	**2 076**
**ENN-B1**	1 822	1 822 000			3 024 520	43 207	**1 296**

** estimated based on our previous study* ***(Fenclova et al., 2019)****, sample No 21, coded as CZ 2.*

Table S2: The fold changes for 92 lipid features filtered by ANOVA. The lipids are ordered by the lipid class and fatty acid chains. The fold changes are expressed as log2(FC), so the breakpoint between downregulation and upregulation is equal to 0. The fold changes over 50% are highlighted in red).

	**Lipid name** @ retention time (min) @ *m/z*	**FC**					
		**B2 over A1**	**C3 over A1**	**D4 over A1**	**B2 over C3**	**B2 over D4**	**D4 over C3**
**CER**	Cer(18:1/16:0)@9.69@596.5242	0.25	-0.03	0.18	0.28	0.07	0.21
	Cer(18:1/23:0)@12.76@694.6332	0.43	0.00	0.22	0.43	0.21	0.22
	Cer(18:2/23:0)@11.99@632.5962	0.41	0.03	0.22	0.38	0.18	0.19
	Cer(25:2/15:0)@11.84@678.6015	0.51	0.05	0.20	0.46	0.31	0.15
**DG**	DG(16:0/18:2)@9.99@610.5401	0.31	0.27	0.41	0.04	-0.10	0.14
	DG(18:1/18:1)@10.56@638.5713	0.37	0.22	0.57	0.15	-0.20	0.35
	DG(18:1/18:2)@10.01@636.5559	0.60	0.36	0.56	0.24	0.04	0.20
	DG(18:1/20:4)@9.82@660.5557	0.68	0.35	0.50	0.33	0.18	0.15
	DG(18:2/18:2)@9.47@634.5400	0.80	0.47	0.64	0.34	0.16	0.17
	DG(18:2/20:1)@10.75@664.5865	0.84	0.28	0.48	0.56	0.36	0.20
	DG(18:2/20:4)@9.18@658.5387	0.97	0.44	0.60	0.53	0.37	0.15
	DMPE(16:0/18:1)@9.65@744.5508	-0.25	-0.07	-0.06	-0.18	-0.19	0.01
**FFA**	FA(18:1)@5.16@281.2481	0.42	0.24	0.22	0.18	0.20	-0.02
	FA(22:5)@4.11@329.2476	1.08	0.45	0.02	0.63	1.06	-0.43
**FAHFA**	FAHFA(16:1/18:3)@3.79@529.4217	0.35	0.16	0.44	0.20	-0.09	0.29
	FAHFA(16:1/20:4)@3.86@555.4373	0.26	0.11	0.35	0.15	-0.09	0.24
	FAHFA(18:0/22:3)@6.28@615.5330	0.32	0.22	0.29	0.10	0.03	0.07
	FAHFA(20:1/22:3)@6.46@641.5474	0.99	0.40	0.34	0.59	0.65	-0.06
	FAHFA(20:4/18:3)@3.64@579.4372	0.04	0.14	0.29	-0.10	-0.25	0.15
	HexCer(18:1/22:1)@10.75@780.6329	-0.46	-0.01	-0.12	-0.45	-0.35	-0.10
	HexCer(22:1/16:0)@10.81@754.6174	-0.36	-0.04	-0.17	-0.32	-0.19	-0.13
**LPC**	LPC(18:0)@3.56@524.3706	0.39	0.16	0.24	0.23	0.15	0.08
	LPC(19:0)@4.56@538.3858	0.58	0.41	0.35	0.17	0.23	-0.06

	LPC(20:4)@2.05@544.3383	0.51	0.49	0.17	0.02	0.35	-0.32
	LPC(22:5)@2.32@570.3545	0.30	0.57	-0.03	-0.27	0.33	-0.60
**PC**	PC(16:0/18:2)@9.20@816.5725	-0.24	-0.06	-0.04	-0.17	-0.20	0.03
	PC(16:0/20:1)@9.97@788.6163	-0.30	-0.15	-0.14	-0.15	-0.16	0.01
	PC(16:0/20:4)@8.51@782.5678	0.03	0.07	-0.04	-0.04	0.07	-0.11
	PC(16:0/22:6)@8.27@806.5675	0.04	0.08	-0.15	-0.05	0.19	-0.24
	PC(18:0/18:1)@10.52@846.6209	-0.33	-0.16	-0.08	-0.17	-0.25	0.08
	PC(18:0/18:2)@9.80@844.6046	-0.15	-0.08	0.00	-0.08	-0.16	0.08
	PC(18:0/20:3)@10.30@870.6196	-0.55	-0.02	0.19	-0.52	-0.73	0.21
	PC(18:0/20:3)@9.57@812.6147	-0.25	-0.11	-0.05	-0.15	-0.21	0.06
	PC(18:0/22:5)@9.96@894.6195	-0.12	0.26	-0.39	-0.38	0.27	-0.65
	PC(18:0/22:6)@9.02@834.5993	0.00	0.02	-0.18	-0.02	0.18	-0.19
	PC(18:1/18:2)@9.34@842.5886	-0.27	-0.01	0.02	-0.25	-0.29	0.03
	PC(18:1/20:3)@8.95@810.5973	-0.12	-0.02	-0.05	-0.11	-0.07	-0.03
	PC(18:1/22:6)@8.57@890.5885	0.10	0.13	-0.16	-0.03	0.27	-0.30
	PC(18:2/20:4)@8.04@864.5729	0.03	0.19	-0.07	-0.15	0.11	-0.26
	PC(18:2/22:6)@7.71@830.5656	0.17	0.36	-0.12	-0.18	0.29	-0.48
	PC(34:3)@8.03@778.5360	0.20	-0.01	0.14	0.21	0.06	0.16
	PC(36:1)@9.95@810.5989	-0.50	-0.27	-0.37	-0.23	-0.13	-0.10
	PC(38:2)@10.08@836.6136	-0.42	-0.21	-0.43	-0.21	0.01	-0.22
	PC(38:6)@7.92@828.5508	0.04	0.19	0.01	-0.16	0.03	-0.18
	PC(40:7)@8.38@854.5664	0.16	0.13	-0.02	0.03	0.19	-0.15
	PC(40:8)@7.71@852.5508	0.15	0.29	-0.10	-0.13	0.25	-0.39
**PE**	PE(16:0/18:2)@8.88@716.5229	-0.18	-0.13	0.11	-0.05	-0.29	0.24
	PE(16:0/18:2)@9.13@714.5057	-0.31	-0.14	0.04	-0.17	-0.35	0.18
	PE(16:0/20:4)@8.92@738.5059	-0.20	-0.06	-0.06	-0.14	-0.14	0.00
	PE(16:0/22:6)@8.65@762.5064	-0.06	0.00	-0.10	-0.06	0.04	-0.10
	PE(18:0/18:1)@10.73@744.5521	-0.10	-0.07	0.16	-0.02	-0.26	0.24
	PE(18:0/18:2)@10.02@742.5370	-0.27	-0.24	0.00	-0.03	-0.27	0.25

	PE(18:0/20:3)@10.22@768.5502	-0.35	-0.27	-0.08	-0.07	-0.27	0.20
	PE(18:0/20:4)@9.85@766.5371	-0.16	-0.08	-0.07	-0.08	-0.08	0.01
	PE(18:0/22:6)@9.57@790.5376	-0.11	-0.14	-0.16	0.02	0.05	-0.03
	PE(18:1/18:2)@9.23@740.5214	-0.09	-0.07	0.10	-0.01	-0.18	0.17
	PE(18:1/20:3)@9.63@766.5366	-0.31	0.02	-0.12	-0.33	-0.19	-0.14
	PE(18:1/20:4)@9.27@764.5214	-0.08	0.18	-0.27	-0.26	0.19	-0.45
	PE(34:3)@8.23@714.5066	0.05	-0.03	0.34	0.08	-0.29	0.37
	PE(36:1)@10.15@746.5685	-0.13	-0.10	0.15	-0.04	-0.28	0.24
	PE(36:2)@9.60@744.5547	-0.09	-0.16	0.12	0.06	-0.22	0.28
	PE(36:3)@8.95@742.5382	0.02	-0.05	0.21	0.07	-0.19	0.25
	PE(36:4)@8.35@740.5220	0.01	0.06	0.32	-0.04	-0.31	0.27
	PE(38:3)@9.75@770.5680	-0.17	-0.15	-0.01	-0.01	-0.16	0.15
	PE(38:5)@9.61@766.5358	-0.02	-0.11	0.17	0.09	-0.19	0.28
	PE(40:6)@9.75@792.5513	-0.24	-0.16	0.07	-0.09	-0.32	0.23
	PI(18:0/20:3)@8.88@887.5625	-0.39	-0.32	-0.08	-0.07	-0.30	0.24
Plasmenyl	plasmanyl-PE(36:5)@9.07@724.5268	-0.12	-0.10	-0.13	-0.02	0.01	-0.04
	plasmanyl-PE(38:5)@9.79@752.5581	0.18	-0.06	-0.12	0.24	0.31	-0.06
	plasmenyl-PE(16:0/20:4)@9.36@722.5106	-0.22	-0.15	-0.29	-0.07	0.08	-0.15
	plasmenyl-PE(16:0/22:4)@10.00@750.5416	-0.34	-0.10	-0.19	-0.24	-0.15	-0.09
	plasmenyl-PE(16:0/22:6)@9.05@746.5101	0.11	-0.08	-0.27	0.19	0.38	-0.19
	plasmenyl-PE(18:0/22:6)@9.95@774.5413	0.33	-0.01	-0.27	0.34	0.60	-0.26
	plasmenyl-PE(18:1/18:1)@10.50@726.5417	0.76	0.08	0.58	0.69	0.18	0.50
	plasmenyl-PE(18:1/20:1)@11.28@754.5729	0.38	-0.12	0.32	0.50	0.05	0.45
	plasmenyl-PE(18:1/22:4)@10.13@776.5571	-0.05	-0.10	-0.22	0.05	0.17	-0.12
	plasmenyl-PE(18:1/22:6)@9.19@772.5261	0.63	0.17	-0.24	0.46	0.87	-0.40
TG	TG(16:0/16:0/18:1)@13.96@850.7850	-0.36	-0.15	0.18	-0.21	-0.54	0.34
	TG(16:0/18:0/18:1)@14.28@878.8157	-0.42	-0.27	0.18	-0.15	-0.60	0.45
	TG(16:0/18:1/22:6)@13.11@922.7855	1.02	0.26	0.28	0.76	0.74	0.02
	TG(16:0/18:1/22:6)@13.28@922.7855	0.87	0.22	0.11	0.65	0.76	-0.10

	TG(16:0/18:2/18:3)@12.91@870.7547	0.20	-0.02	0.29	0.22	-0.09	0.31
	TG(16:0/18:2/22:6)@12.93@920.7694	0.82	0.11	0.11	0.70	0.70	0.00
	TG(16:0/18:3/18:3)@12.57@868.7386	0.34	-0.04	0.33	0.38	0.01	0.37
	TG(16:0/18:3/18:3)@12.83@868.7386	0.07	-0.09	0.39	0.17	-0.32	0.49
	TG(16:1/16:1/18:2)@12.86@844.7389	-0.08	-0.08	0.43	0.01	-0.50	0.51
	TG(17:0/18:2/18:2)@13.44@886.7852	0.35	0.14	0.35	0.21	0.00	0.21
	TG(18:1/18:1/20:1)@14.25@930.8480	0.51	0.09	0.38	0.42	0.13	0.29
	TG(18:1/18:2/18:3)@12.91@896.7699	0.56	0.26	0.20	0.29	0.36	-0.06
	TG(18:1/18:2/20:1)@14.01@928.8291	0.62	0.10	0.27	0.52	0.35	0.17
	TG(18:1/18:3/20:1)@13.81@926.8165	0.47	0.08	0.17	0.39	0.30	0.09
	TG(18:2/18:2/18:3)@12.78@894.7538	0.42	0.02	0.23	0.41	0.19	0.22

Table S3: The effect of mycotoxin exposure on hepatic lipid profile.

Data are from binary OPLS-DA models with VIP score > 1; B2 vs. A1 (38 lipids)

- higher intesity in B2 (mycotoxin-exposed mice)

- higher intesity in A1 (control mice)

OPLSDA B2 vs. A1	
Lipid name	VIP score
DG(18:2/20:4)	1.83
FAHFA(20:1/22:3)	1.79
TG(16:0/18:1/22:6)	1.78
FA(22:5)	1.76
TG(16:0/18:1/22:6)	1.73
DG(18:2/18:2)	1.72
DG(18:2/20:1)	1.70
TG(16:0/18:2/22:6)	1.70
plasmenyl-PE(18:1/18:1)	1.57
LPC(19:0)	1.55
DG(18:1/18:2)	1.46
TG(18:1/18:2/20:1)	1.44
DG(18:1/20:4)	1.43
TG(18:1/18:1/20:1)	1.34
TG(18:1/18:2/18:3)	1.34
Cer(25:2/15:0)	1.33
LPC(20:4)	1.30
Cer(18:1/23:0)	1.29
Cer(18:2/23:0)	1.20

TG(18:1/18:3/20:1)	1.19
TG(18:2/18:2/18:3)	1.18
FA(18:1)	1.17
LPC(18:0)	1.17
PEP(18:1/22:6)	1.08
FAHFA(16:1/18:3)	1.06
DG(18:1/18:1)	1.06
TG(17:0/18:2/18:2)	1.02
FAHFA(16:1/20:4)	1.01
PC(36:1)	1.36
PC(18:0/20:3)	1.29
PC(38:2)	1.19
plasmenyl-PE(16:0/22:4)	1.18
PE(18:0/20:3)	1.15
HexCer(18:1/22:1)	1.14
TG(16:0/18:0/18:1)	1.13
PI(18:0/20:3)	1.07
PC(18:0/18:1)	1.07
PE(16:0/18:2)	1.02

Table S4: The effect of mycotoxin plus silymarin exposure on hepatic lipid profile.

Data are from binary OPLS-DA models with VIP score > 1; C3 vs. A1 (29 lipids)

- higher intesity in C3 (silymarin-treated mice)

- higher intesity in A1 (control mice)

OPLSDA C3 vs. A1	
Lipid name	VIP score
LPC(22:5)	2.26
LPC(19:0)	1.96
DG(18:2/20:4)	1.94
DG(18:2/18:2)	1.94
FA(22:5)	1.85
PC(18:2/22:6)	1.80
LPC(20:4)	1.77
DG(18:1/20:4)	1.75
PC(40:8)	1.73
FAHFA(20:1/22:3)	1.73
DG(18:1/18:2)	1.65
DG(16:0/18:2)	1.51
DG(18:2/20:1)	1.41
FA(18:1)	1.32
FAHFA(18:0/22:3)	1.31
TG(18:1/18:2/18:3)	1.23
PC(38:6)	1.22
PC(18:0/22:5)	1.22

PC(18:2/20:4)	1.15
TG(16:0/18:1/22:6)	1.14
DG(18:1/18:1)	1.07
PI(18:0/20:3)	1.61
PE(18:0/20:3)	1.57
PE(18:0/18:2)	1.53
PC(36:1)	1.51
TG(16:0/18:0/18:1)	1.34
PC(38:2)	1.16
PE(36:2)	1.12
PE(38:3)	1.04

Table S5: The effect of mycotoxin plus silymarin exposure on hepatic lipid profile.

Data are from binary OPLS-DA models with VIP score > 1; D4 vs. A1 (33 lipids)

- higher intesity in D4 (mycotoxin plus silymarin-exposed mice)

- higher intesity in A1 (control mice)

OPLSDA D4 vs. A1	
Lipid name	VIP score
DG(18:2/18:2)	2.11
DG(18:1/18:1)	1.99
DG(18:1/18:2)	1.97
DG(18:2/20:4)	1.89
DG(18:1/20:4)	1.75
plasmenyl-PE(18:1/18:1)	1.74
DG(18:2/20:1)	1.67
DG(16:0/18:2)	1.62
FAHFA(16:1/18:3)	1.58
FAHFA(16:1/20:4)	1.52
TG(16:0/18:3/18:3)	1.51
TG(16:1/16:1/18:2)	1.43
TG(17:0/18:2/18:2)	1.43
LPC(19:0)	1.42
PE(34:3)	1.38
PE(36:4)	1.35
TG(18:1/18:1/20:1)	1.34
FAHFA(18:0/22:3)	1.28

TG(16:0/18:3/18:3)	1.28
FAHFA(20:4/18:3)	1.28
TG(16:0/18:2/18:3)	1.21
LPC(18:0)	1.09
plasmenyl-PE(18:1/20:1)	1.07
PE(36:3)	1.07
TG(18:1/18:2/20:1)	1.02
TG(16:0/18:1/22:6)	1.02
PC(38:2)	1.57
PC(36:1)	1.43
PC(18:0/22:5)	1.26
plasmenyl-PE(16:0/22:6)	1.16
plasmenyl-PE(18:0/22:6)	1.12
plasmenyl-PE(16:0/20:4)	1.05
PE(18:1/20:4)	1.01

Figure S1: Liver steatosis staging: A - score 0 (steatosis of less than 5 % of liver cells), B - score 1 (steatosis of up to 33 % of liver cells), C - score 2 (steatosis of up to 66 % of liver cells), D - score 3 (steatosis of more than 66 % of liver cells).

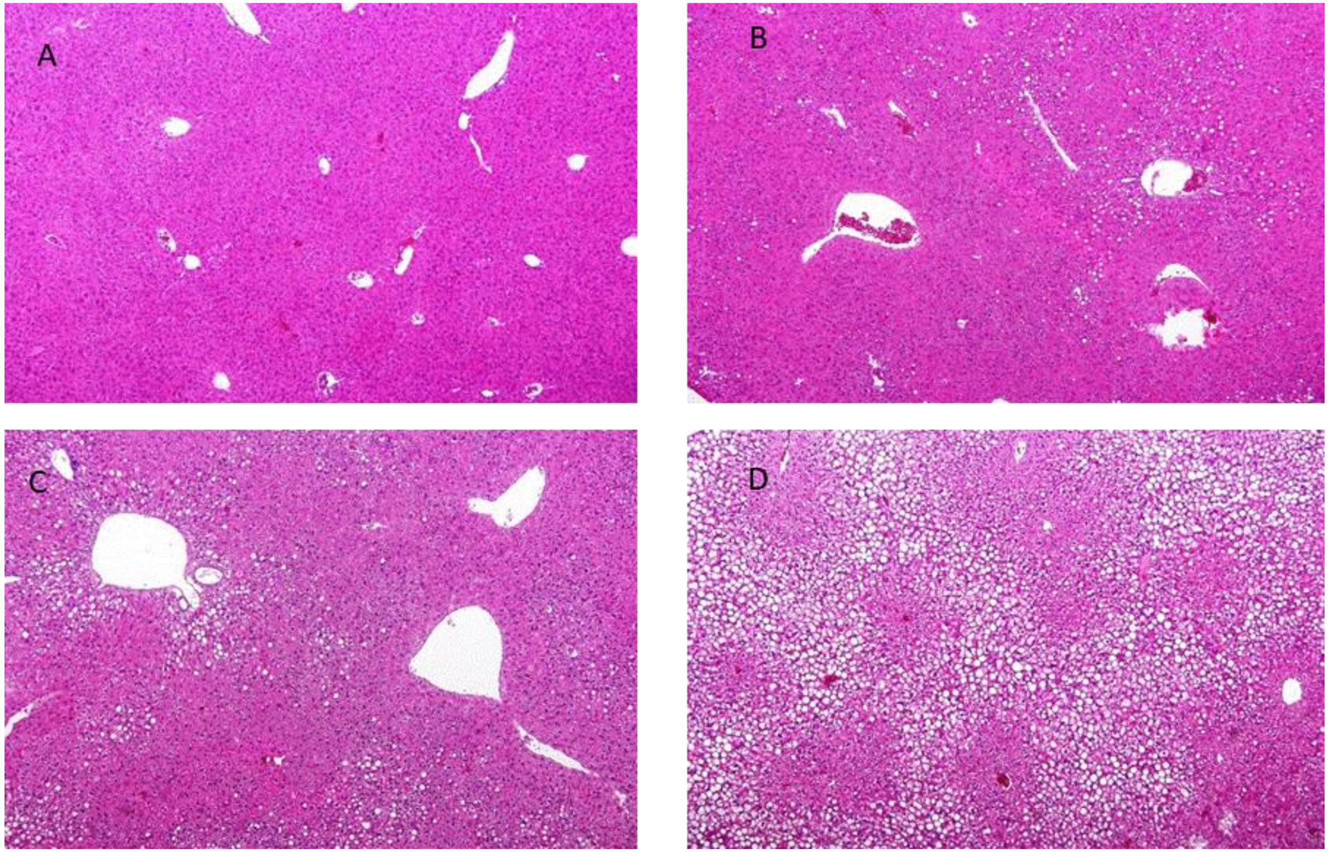

Figure S2: Boxplots for all 28 significant lipids with OPLS-DA VIP score >1, significantly contributing to group separation in at least two binary comparisons.

DG:

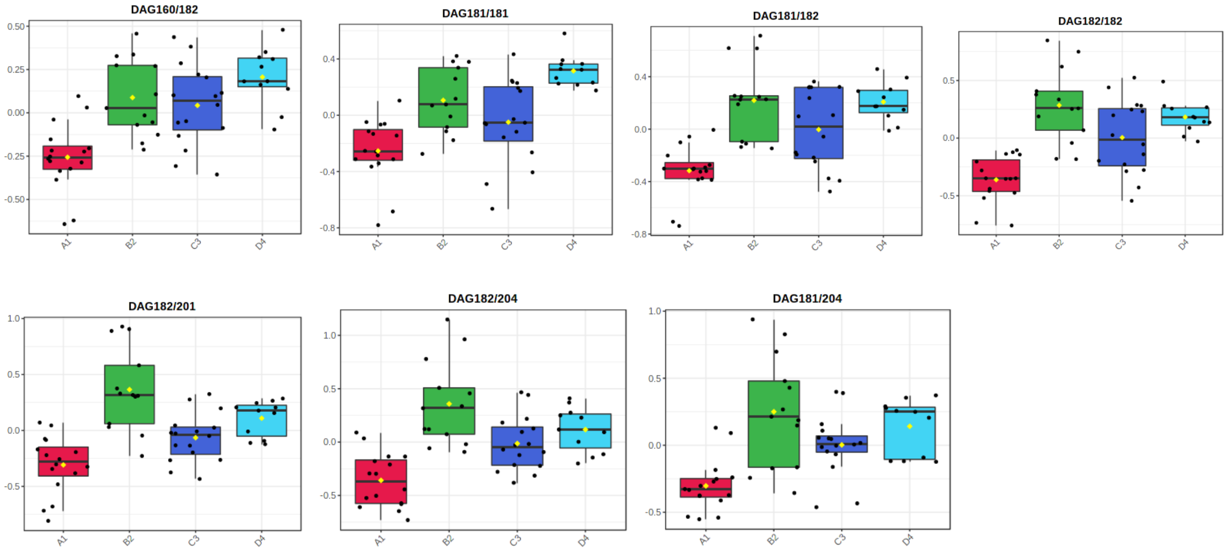

FA:

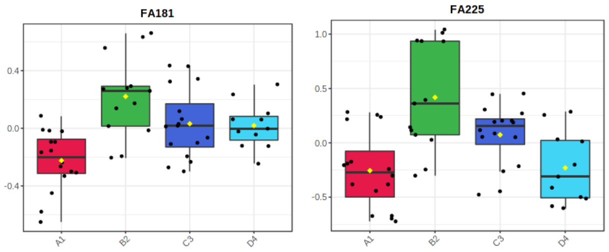

FAHFA:

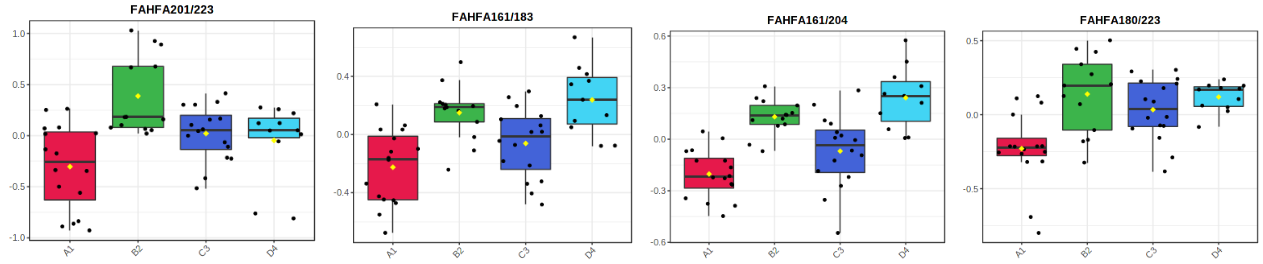
FAHFA201/223
FAHFA161/183
FAHFA161/204
FAHFA180/223
A1
B2
C3
D4

LPC:

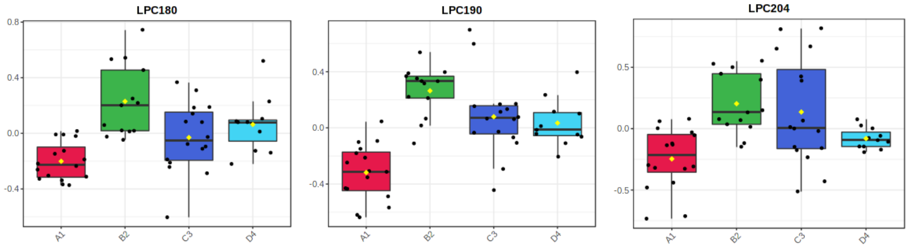
LPC180
LPC190
LPC204
A1
B2
C3
D4

PL:

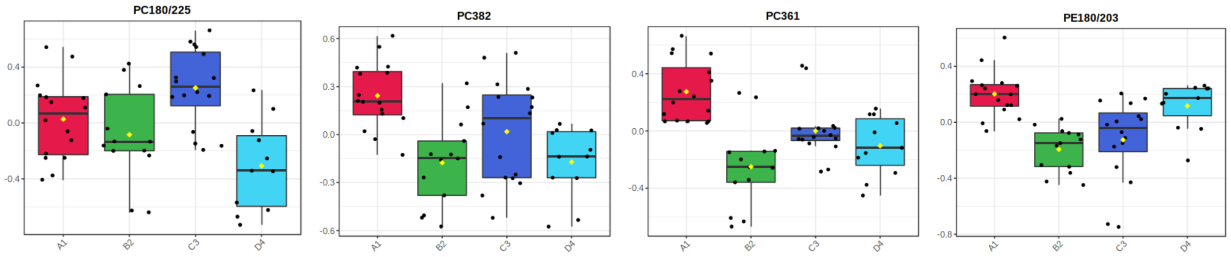
PC180/225
PC382
PC361
PE180/203
A1
B2
C3
D4

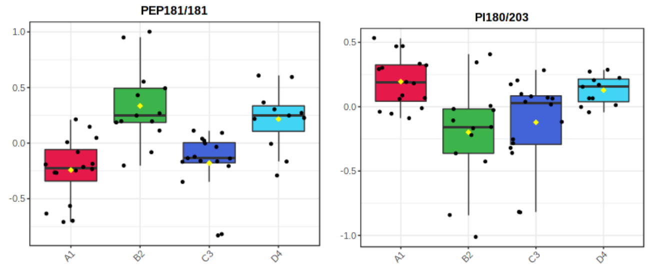
PEP181/181
PI180/203
A1
B2
C3
D4

TG:

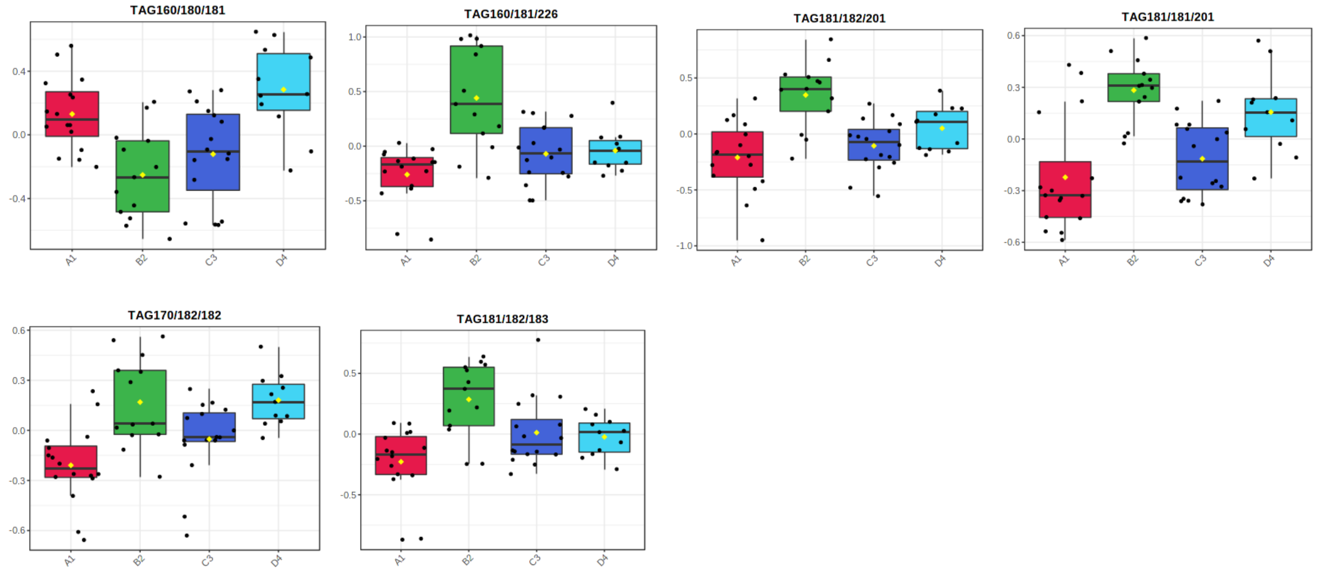
TAG160/180/181
TAG160/181/226
TAG181/182/201
TAG181/181/201
TAG170/182/182
TAG181/182/183
A1
B2
C3
D4
